# 

**Published:** 1876-08

**Authors:** 


### Books and Pamphlets Received.

---

A Treatise on Surgery. Its principles and practice. By T. Holmes, M. A., Cantab. Philadelphia: Henry C. Lea, 1876.

A Treatise on the Science and Practice of Midwifery. By W. S. Playfair, M. D., F. R. C. P. Philadelphia: Henry C. Lea, 1876.

A Practical Treatise on Diseases of the Eye. By Robert Brudenell Carter, F. C. S. Edited with additions and Test Types. By John Green, M. D. Philadelphia: Henry C. Lea, 1876.

The Pathology and Treatment of Child-Bed. A Treatise for Physicians and Students. By Dr. F. Winckel. Translated from the second German edition by James R. Chadwick, M. D. Philadelphia: Henry C. Lea, 1876.

Lectures on Fever. Delivered in the Theatre of the Meath Hospital and County of Dublin Infirmary. By William Stokes, M. D., D. C. L., Oxon., F. R. S. Edited by John William Moore, M. D., F. K. Q. C. P. Philadelphia: Henry C. Lea, 1876.

An Introduction to Pathology and Morbid anatomy. By T. Henry Green, M. D , Lond. Second American, from the third English Edition. Philadelphia: Henry C. Lea, 1876.

A Manual of Percussion and Auscultation; of the Physical Diagnosis of Diseases of the Lungs and Heart and Thoracic Aneurism. By Austin Flint, M. D. Philadelphia: Henry C. Lea, 1876.

Theory of Medical Science. By William R. Dunham, M. D. Boston: James Campbell, 1876.

Chirurgie Antiseptigue, Principes Modes d'application et Resultats du Pansement de Lister. Par Le Dr. Just Lucas-Championniére. Avec figures dans le texte. Paris Librairie: J. B. Baillière et Fils, 1876.

An Address on some of the Leading Publie Health Questions; with remarks on the extent of swamp lands in the United States and their reclamation as a Sanitary Measure. Delivered at the opening of the Third Annual Meeting of the American Public Health Association. By J. M. Toner, M. D., President. Reprint from Vol. II, Public Health Papers. Cambridge: The Riverside Press, 1876.

Chorea. Its Causes and Treatment. By George T. Stevens, M. D. New York: D. Appleton & Co., 1876. Reprint from the transactions of the New York Academy of Medicine.

Proceedings of the Medical Society of the County of Kings, Brooklyn, N. Y., August, 1876.

Transactions of the Medical Society of the District of Columbia. July, 1876.

A Clinical lecture on the Treatment of Incipient Stricture, by Otis's Operation. By Mr. Berkley Hill, together with explanatory remarks on the Treatment of Stricture and Gleet. By Fessenden A. Otis, M. D. From the "Lancet" of April 8th and June 3d and 10th, 1876.

